# Designing of improved drugs for COVID-19: Crystal structure of SARS-CoV-2 main protease M^pro^

**DOI:** 10.1038/s41392-020-0178-y

**Published:** 2020-05-09

**Authors:** Hylemariam Mihiretie Mengist, Xiaojiao Fan, Tengchuan Jin

**Affiliations:** 1 Department of Obstetrics and Gynecology, The First Affiliated Hospital of USTC, Division of Life Sciences and Medicine, University of Science and Technology of China, 230001 Hefei, Anhui China; 2 Laboratory of Structural Immunology, CAS Key Laboratory of Innate Immunity and Chronic Disease, Division of Life Sciences and Medicine, University of Science and Technology of China, 230027 Hefei, Anhui China

**Keywords:** Structural biology, Infectious diseases

**Recently, Zhang and colleagues published a study in** ***Science*** **which reports about the crystal structure of the main protease (M**^**pro**^**, 3CL**^**pro**^**) of SARS-CoV-2**^1^. **The authors reported the X-ray structure of unliganded SARS-CoV-2 M**^**pro**^ **and its complex with α-ketoamide inhibitor after a modification from the previously designed inhibitor through incorporating P3-P2 amide bond into pyridone ring so as to increase its half-life in plasma. Finally the authors demonstrated that the crystal structure of M**^**pro**^ **provides a basis for designing of a potent inhibitor to the protease with a marked tropism to the lung and with ease of administration through inhalation**.

An outbreak of series of acute respiratory illness caused by a novel coronavirus, SARS-CoV-2, caused a global threat in 2020^2^. The world health organization (WHO) named the disease “COVID-19” and declared it as a world health emergency pandemic. Studying the crystal structure of targets for treatment is very crucial to know the mechanism of action of prospective drugs. A structural study on the coronavirus main protease 3CL^pro^ inhibitor complex established designing of broad-spectrum halomethyl ketone inhibitors to the Coronaviridae family and demonstrated that these inhibitors form a thioether linkage with high affinity to the target^3^. Hilgenfeld’s group reported earlier that a structure-based design of peptidomimetic α-ketoamides are also effective broad-spectrum inhibitors to the main and 3C protease of coronaviruses and enteroviruses as demonstrated by crystal structure of inhibitor-protease complex^4^. The 3CL protease of coronaviruses facilitates viral assembly by cleaving polyproteins and most active compounds prevent disease progression by inhibiting viral proteases ^5^.

In their study, Zhang et al. modified the previously designed best inhibitor (**11r**) to increase its half-life in plasma, increase its solubility and reduce its binding to plasma proteins^1^. Here, the authors hide the P2-P3 amide bond into a pyridone ring to prevent it from cleavage by cellular proteases so that its plasma half-life is increased. To increase solubility of the inhibitor and reduce its binding to plasma proteins, the authors replaced the hydrophobic cinnamoyl moiety with a less hydrophobic Boc group.

The introduced pyridone ring should be compatible with the three-dimensional structure of the target which has a crucial role for effective inhibition. In order to confirm this, the authors solved the crystal structure of M^pro^ of SARS-CoV-2 at 1.75 Å resolution and found that the crystal structure is highly similar to that of SARS-CoV M^pro^ only with a 0.53 Å r.m.s difference between the two free enzymes. SARS-CoV-2 M^pro^ forms a tight dimer and has a contact interface mainly between domain II of molecule A and the NH_2_-terminal residues of molecule B where this dimerization is important for catalytic activity. Unlike SARS-CoV-2, SARS-CoV M^pro^ dimer has a polar interaction between the two domains III involving a 2.60 Å hydrogen bond between the side chain hydroxyl groups of Thr^285^ of each protomer which is also supported by a hydrophobic interaction between the side chain of Ile^286^ and Thr^285^. In SARS-CoV-2 M^pro^, threonine is replaced with alanine and isoleucine with leucine. As suggested by authors, Alanine replacements change the enzyme dynamics and increase its catalytic activity by allowing the two domains III to be in a close contact; although, the catalytic activity of SARS-CoV-2 M^pro^ was only slightly higher with similar *K*_d_ of dimer dissociation (~2.5 μM).

The authors named the modified α-ketoamide inhibitor **13a**. Compared to **11r**, **13a** has a 3 fold increase in plasma half-life in mice, highly soluble in plasma with a 19 fold increase in solubility in-vitro and a 13 fold increase in thermodynamic solubility. Further the binding of **13a** with plasma proteins was reported to decrease to 97% as compared to **11r** which showed 99% binding activity with plasma proteins. Provided these improvements, the structural modification resulted in loss of some inhibitory activities against the main protease of SARS-CoV-2 and 3C proteases of enteroviruses as evidenced by increased IC_50_ value.

The authors increase the antiviral activity of **13b** against beta coronaviruses of clade b by sacrificing the broad-spectrum nature of **13a** through replacing the P2 cyclohexyl moiety in **13a** with a small cyclopropyl in **13b**. The authors determined the X-ray structure of α-ketoamide **13b** and SARS-CoV-2 M^pro^ complex at 1.95 and 2.20 Å resolution. In one of the structures, the authors found that the key residue, Glu^166^, forms inactive conformation in protomer B while the **13b** compound is bound in the same fashion as in molecule A. Authors also found that the inhibitor binds to the shallow substrate-binding site at the surface of protomers between domains I and II in the inhibited SARS-CoV-2 M^pro^.

**Fig. 1:**
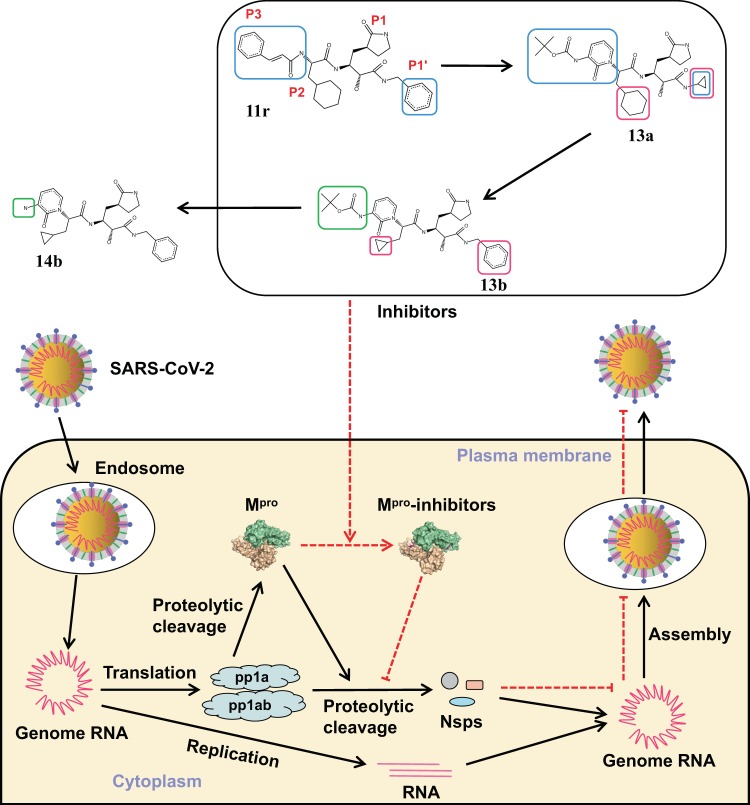
Inhibitors of protease M^pro^ prevent the replication of SARS-CoV-2. After entering into the host cell, SARS-CoV-2 releases its genomic RNA. The process of translation yields polyproteins pp1a and pp1ab, which are cleaved to the main protease M^pro^ and nonstructural proteins (nsps). M^pro^ is involved in the producing of nsps. Nsps is essential for assembling the viral replication transcription complex (RTC) to engage in RNA synthesis. Once inhibitors, such as **11r**, **13a**, and **13b**, act in the cell, they bind to M^pro^ and inhibit the activity of this enzyme, resulting in failure of virion assembly. Ultimately, host cell fails to release the new intact virions. Colored box highlight the modifications from **11r** to **14b**. Due to lacking Boc group, **14b** could not cross the cellular membrane to inhibit viral replication

The Boc group was removed in compound **14b** resulting in inactive form which means the Boc group is necessary to cross the cellular membrane and even more hydrophobic moiety may be advantageous provided that it may result in increased plasma protein binding. Hilgenfeld’s group assessed the pharmacokinetics of **13a** and **13b** and found that the absorption-distribution-metabolism-excretion of both compounds was similar with a 90% binding to human plasma proteins. The **13b** showed less clearance compared to **13a** and good tropism to the lungs with a well-tolerated administration through inhalation in mice. Taken together, the authors designed α-ketoamide inhibitors with improved inhibition efficiency against recombinant SARS-CoV-2 M^pro^ to suppress disease progression (Fig. 1). Their work provides insights into the development of more potent pyridone containing anti-coronaviral drugs. Future structural-functional studies are recommended to improve the efficacy of targeted therapies to tackle the emerging pandemics due to coronaviruses.
